# TEACHING AGING WITH A GLOBAL CLASSROOM

**DOI:** 10.1093/geroni/igae098.2665

**Published:** 2024-12-31

**Authors:** Kristine Mulhorn

**Affiliations:** Drexel University, Philadelphia, Pennsylvania, United States

## Abstract

In a time of heightened interest in teaching about aging in undergraduate programs, Drexel launched a competitive effort to offer courses that would be of interest across a variety of majors and minors. As a scholar working in aging in Japan for several years, as well as a background in aging policy, I launched a course called “Aging, Aging Policy and Health in Asia”. The highlight was the global classroom—a three-week project between Drexel students and students at KAIST (Korean Advanced Institute of Science and Technology) on the topic of aging and technology. The focus of the effort to teach aging across the university was to assure that the AGHE competencies were included in these courses. We included competencies such as the bio-psycho-social model for healthy aging, the epidemiologic transition, and the ethics of caregiving. This course resulted in close collaboration related to a product or concept that had potential for direct application In the lives of older adults. For example, one project addressed the use of research and technology applied to aging policies of Korea and the US, and another looked closely at strategies that improve intergenerational interactions that improve the lives of older adults and children. This presentation will demonstrate the integration of AGHE competencies into the course, as well as the ways the students met the learning objectives within the course.

